# The Function of Microglia in Cognitive Impairment Influenced by Sleep Deprivation

**DOI:** 10.1007/s10571-025-01654-x

**Published:** 2026-01-14

**Authors:** Jiping Jiang, Min Li, Yulong Xia, Wei Wei, Sheng Li, Xiao Wu, Sen Li, Houping Xu

**Affiliations:** 1 Geriatric Department, The Affiliated Traditional Chinese Medicine Hospital, Southwest Medical University, Luzhou, Sichuan China; 2 Division of Spine Surgery, Department of Orthopedic Surgery, Nanjing Drum Tower Hospital, Affiliated Hospital of Medical School, Nanjing University, Nanjing, Jiangsu China; 3 The Affiliated Traditional Chinese Medicine Hospital, Southwest Medical University, Luzhou, Sichuan, China; 4 Acupuncture and Rehabilitation Department, The Affiliated Traditional Chinese Medicine Hospital, Southwest Medical University, Luzhou, Sichuan, China

**Keywords:** Microglia, Sleep deprivation, Cognitive impairment, Function

## Abstract

Sleep deprivation, resulting from factors such as lifestyle, disease, or environmental influences, directly contributes to cognitive decline. Research has found that the impact of sleep deprivation on microglia may be a key factor in cognitive impairment. The specific mechanisms through which microglia contribute to this process are not yet fully understood. It may act through multiple pathways, including the accumulation of excitatory neurotransmitters, Aβ plaque deposition, neuroinflammation, disrupted autophagy, abnormal cell death, and impaired synaptic plasticity. This review synthesizes evidence from the past two decades on the interplay between microglia, sleep deprivation, and cognitive impairment. It provides a comprehensive overview of associated factors and their operational pathways, analyzes the network of pathological interactions, and identifies possible treatment directions. It also emphasizes the dual functions of microglia in worsening and alleviating cognitive impairment while investigating possible therapeutic strategies targeting microglial function. This review aims to clarify microglial pathways in sleep-loss-related cognitive deficits, thereby advancing the field and providing a foundation for new therapeutic strategies.

## Introduction

**Fig. 1:**
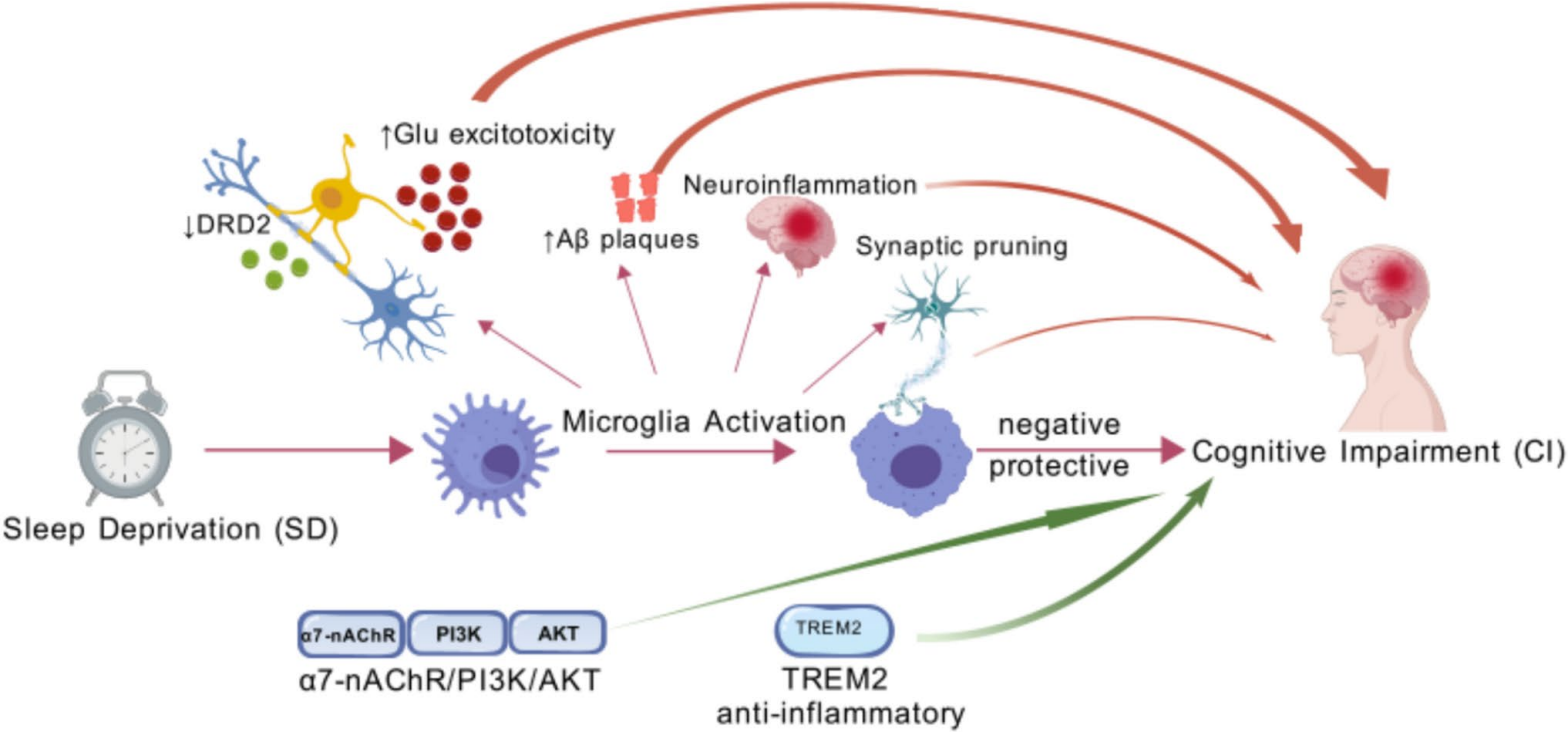
Microglia-mediated pathways in sleep deprivation-induced cognitive impairment. Sleep deprivation triggers microglial activation, leading to neuroinflammation, glutamate excitotoxicity, Aβ plaque accumulation, and synaptic pruning via pathways like CX3CR1 and TREM2. These processes impair cognition, while protective mechanisms such as α7-nAChR/PI3K/AKT and TREM2 signaling counteract damage, highlighting microglia’s dual role in cognitive dysfunction

In recent years, growing work pressure, the pervasive use of electronic devices, and the general acceleration of the pace of social life have jointly led to a significant reduction in people’s voluntary sleep time, thus giving rise to the prevalent phenomenon of sleep deprivation (SD). SD broadly describes a state of insufficient sleep, including acute SD, chronic sleep deprivation (CSD), sleep fragmentation (SF), and various sleep restrictions. It may induce numerous physical, mental, and behavioral disorders, with cognitive impairment (CI) being one of the most significant outcomes. This situation has become extremely common in modern society, and the accompanying increase in CI cases poses a significant challenge. Currently, no effective treatment is available (Kouhestani et al. 2018; Wang et al. 2021). Microglia, derived from erythromyeloid progenitors that migrate to the brain and undergo proliferation and branching, represent one of the most crucial immune cell populations within the brain parenchyma (Ginhoux and Prinz 2015). There is a strong connection between activated microglia, neuroinflammation, and CI in neurodegenerative disorders, including Parkinson’s disease (PD), Alzheimer’s disease (AD), and multiple sclerosis (Glass et al. 2010; Graeber et al. 2011). SD likely impairs cognitive function partly by affecting microglia, though the precise mechanisms remain unclear (Fig. 1).

Most existing reviews either focus on the role of microglia in the central nervous system (Graeber et al. 2011) or discuss the sleep-cognition interplay in a broader sense (Gao et al. 2023b; Ji et al. 2021; Yakovleva et al. 2019). A dedicated and systematic review that specifically focuses on the mechanistic role of microglia as a central mediator linking different patterns of SD (e.g., CSD vs. SF) to distinct aspects of CI is still lacking. This represents a significant gap in the literature, as understanding microglia-specific mechanisms is crucial for developing targeted therapies. Our review offers a novel perspective by framing microglia as active drivers of CI, detailing how specific sleep loss patterns alter microglial states and functions. This review aims to synthesize evidence for a precise cause-effect pipeline linking sleep patterns to cognitive deficits via specific microglial actions and lay a solid theoretical foundation for the development of new treatment methods.

A systematic literature search was conducted to identify relevant studies investigating the role of microglia in SD-induced CI. The databases PubMed and Web of Science were searched from their inception up to 2024 using the search string: (sleep deprivation) AND (microglia) AND (cognitive impairment). References from retrieved articles were also manually screened to identify additional potential studies. After combining results from both databases, duplicate records were removed. The study selection was performed independently by two investigators based on the following criteria. Inclusion criteria included study types: randomized controlled trials (RCTs), observational studies, reviews, and clinical studies; population: interventional studies involving sleep-deprived humans or mice; publication type: peer-reviewed journal articles; and language: English only. Exclusion criteria included non-English publications, studies with unavailable full text, retracted articles, studies with low quality (e.g., extremely small sample size, unclear methodology), articles irrelevant to the research topic, editorials, and conference abstracts. To minimize bias, the processes of literature screening and data extraction were carried out independently by two researchers. Any discrepancies were resolved through discussion or by consultation with a third reviewer. As the included studies were predominantly based on preclinical animal models, a formal risk of bias assessment using standardized tools was not performed. However, during the interpretation of the results, the methodological quality of the primary studies, including aspects such as sample size, implementation of blinding, and statistical methods, was critically evaluated to ensure a robust synthesis of the findings. In addition, reference tracing was conducted to minimize retrieval bias.

Notably, current research relies predominantly on preclinical rodent models due to technical and ethical constraints in obtaining human CNS tissue. While this review synthesizes critical mechanistic insights from these models, we explicitly address strategies to bridge specific gaps in the Discussion section.

## The Negative Role of Microglia in SD-Induced CI

### Effect of the Conduction of Neurotransmitters

Microglia express a variety of receptors for neurotransmitters and neuroactive compounds, including glutamate, GABA, dopamine, adrenaline, acetylcholine, adenosine, and ATP receptors (Czapski and Strosznajder 2021).

Dopamine receptor D2 (DRD2), one of the five subtypes of dopamine receptors, plays a crucial role in bridging cognitive function and synaptic plasticity (Iino et al. 2020). It is known that DRD2 is partially expressed by microglia. Multiple studies have shown that SD reduces DRD2 expression in the human brain’s hippocampus (Gao et al. 2023a), caudate nucleus, putamen (Volkow et al. 2008), and ventral striatum (Volkow et al. 2012b), a change that attenuates dopamine signal transduction (Volkow et al. 2012a).

It is known that impaired dopamine signaling results in working memory and executive functioning deficits (Owen et al. 1997). However, whether DRD2 downregulation happens specifically in microglia after SD, and how such a change in this cell type leads to cognitive deficits, is not yet known. Future studies employing techniques such as microglia-specific DRD2 knockout or in vivo imaging are needed to directly establish the causal link between microglial DRD2 signaling and cognitive deficits following SD.

**Fig. 2:**
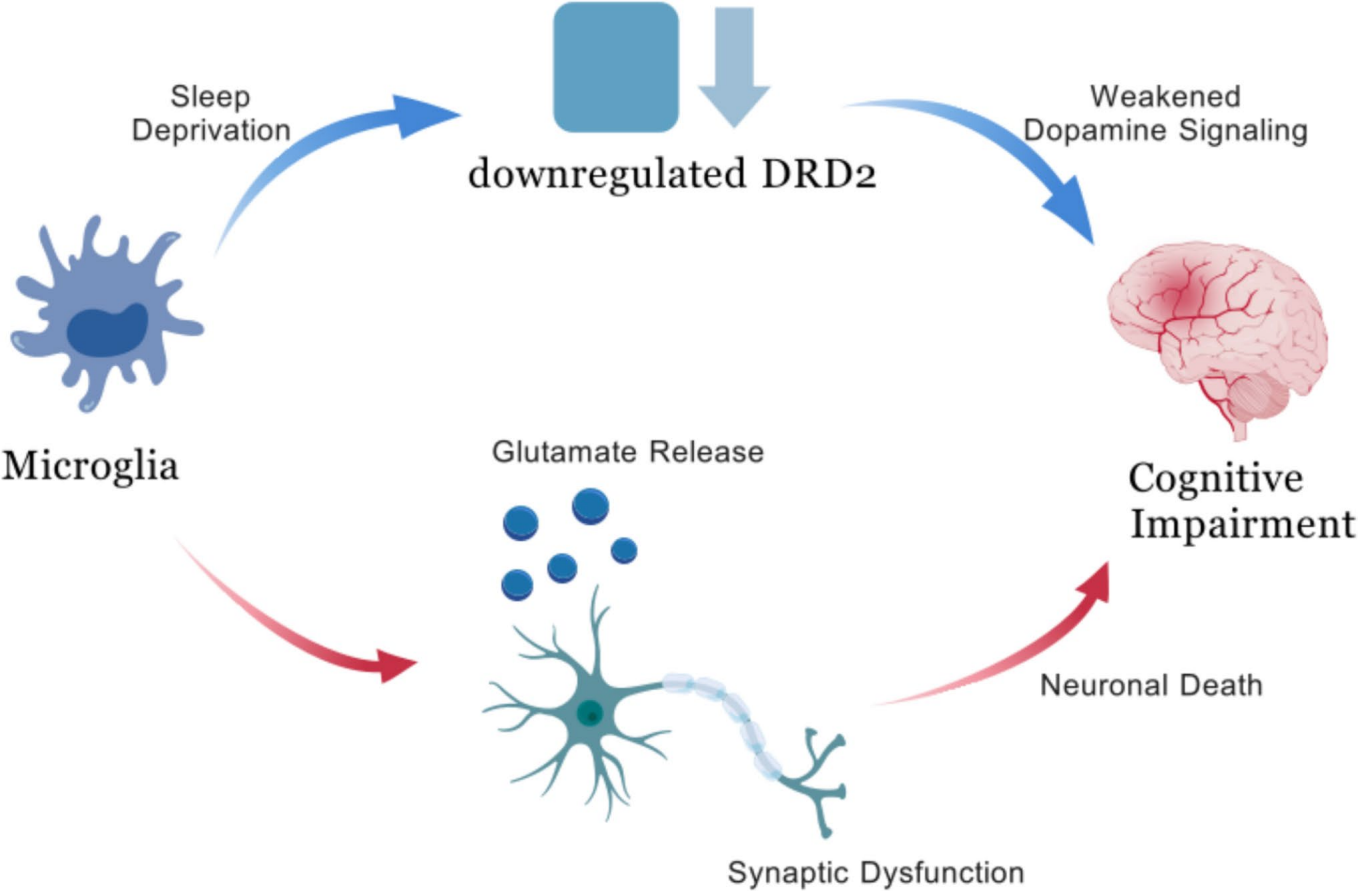
Impact of SD on microglia-mediated CI via dopamine and glutamate pathways. SD downregulates dopamine receptor D2 (DRD2) in microglia, weakening dopamine signaling. This disruption leads to excessive glutamate release from microglia, contributing to neuronal death, synaptic dysfunction, and ultimately CI

Conversely, dopamine inhibits the activation of the NLRP3 inflammasome in microglia, thereby decreasing the release of pro-inflammatory cytokines (Pike et al. 2022). DRD2 agonists can decrease microglial proinflammatory cytokine production through the Cryab/NF-κB pathway, representing a potential treatment strategy for SD-induced CI (Gao et al. 2023a). This suggests another way microglia might contribute to SD-related CI: by downregulating DRD2, their ability to suppress inflammasome activation weakens, which can increase neuroinflammation and impair cognition (Fig. 2).

### Induction of Accumulative Neurotoxicity of Excitatory Neurotransmitters

Microglia express receptors for many neurotransmitters, but for most, their role in SD-related CI is not well understood. Currently, a large body of evidence suggests that glutamate may be involved in this pathological process (Dantzer and Walker 2014; Haroon et al. 2017; He et al. 2024; Reus et al. 2018). Therefore, this review will focus on the role of glutamate.

Glutamate, the primary excitatory neurotransmitter, plays an important role in the CNS. SD induces the excessive activation of microglia. Emerging evidence indicates that activated microglia secrete glutamate (along with ATP), triggering downstream effects via activation of diverse receptor-mediated signaling pathways, including purinergic receptors, such as P2X7 and P2X4 (Illes et al. 2020; Sánchez-Melgar et al. 2019).

The excessive release of glutamate causes hyperactivation of both presynaptic and postsynaptic glutamate receptors, including ionotropic types (kainate, AMPA, and NMDA) and metabotropic subtypes—group I (mGluR1,5), group II (mGluR2,3), and group III (mGluR4,6,8) (Stolero and Frenkel 2021; Zhang et al. 2020). Research suggests that extra synaptic glutamate receptors may play a significant role in neurodegenerative processes by inducing Tau protein expression and suppressing the transcription process of the cAMP-response element binding (CREB) protein (Liu et al. 2019).

Overactivation of microglia results in excessive glutamate release and impaired uptake, triggering excitotoxicity, disrupted extra synaptic communication, synapse loss, neuronal degeneration, and behavioral deficits (Haroon et al. 2017), which are the potential therapeutic targets for glutamate-related neurodegenerative diseases.

Additionally, activated microglia can shift tryptophan metabolism towards the kynurenine (KYN) pathway, resulting in elevated KYN levels. This alteration has been linked to CI-related disorders, including depression, Huntington’s disease, autism, and schizophrenia, as well as other molecules that modulate glutamatergic signaling, such as increased quinolinic acid (QUIN) (Garrison et al. 2018). QUIN exhibits neurotoxic effects that may act additively or synergistically with neuroinflammation or excitotoxicity, further triggering gliotoxicity and neuronal cell death (Ferreira et al. 2020; Guillemin 2012; Schwarcz 2016).

### Triggering of Deposition-Induced Neurological Dysfunction of Amyloid-β Protein (Aβ)

Disruptions in the sleep–wake cycle alter the clearance of Aβ in interstitial fluid and cerebrospinal fluid, leading to its accumulation, a hallmark of AD. CSD exacerbates the deposition of Aβ plaques and tau tangles, both of which are defining pathological features of AD, the most prevalent form of dementia caused by neurodegeneration (Holth et al. 2019; Kang et al. 2009).

Along with Aβ plaques, microgliosis is a widely recognized hallmark pathological feature shared by multiple neurodegenerative disorders, including AD, and acts as a pivotal regulator during the initial phases of AD progression (Long and Holtzman 2019; Ransohoff 2016). While the exact cause of AD remains unclear, current evidence suggests that it is linked to the generation and extracellular buildup of Aβ peptides, as well as the intracellular accumulation of toxic tau aggregates. These processes lead to neurotoxicity, synaptic loss, and eventually CI (Twarowski and Herbet 2023).

In their resting state, microglia exhibit a highly branched structure and minimal activity. However, exposure to stressors like SD can trigger their activation, leading to significant alterations in both form and function. Parhizkar et al. found that SD induces heightened lysosomal activity in microglia, accompanied by a decrease in branch number per cell, enlarged cell soma size in the cortex, and reduced branch lengths. However, microglia fail to engulf Aβ plaques and degrade material due to the rising metabolic demands of cells, which could accelerate Aβ plaque deposition (Parhizkar et al. 2023).

C. Wang’s study also demonstrates that the APOE4 genotype synergizes with SD to impair the microglial response to the amassing of Aβ to exacerbate Aβ plaque deposition and NP-tau pathology along with educed microglial aggregation around plaques with a concomitant rise in dystrophic neurite formation (Wang et al. 2023a). These are all closely related to CI (Sadleir and Vassar 2023). SD promotes amyloid plaque accumulation (Lim et al. 2014), and these findings may subsequently trigger the activation of microglia (Wadhwa et al. 2017; Xiang et al. 2006).

### Neuroinflammation

Long-term SD can cause sustained neuroinflammation, which damages synapses, disrupts neuronal firing, leads to cell death, and hinders the birth of new neurons (Liew and Aung 2021). Research indicates that neuroinflammatory responses, along with microglial proliferation and activation, may lead to impairments in spatial learning, the development of depressive symptoms, and memory consolidation (Yang et al. 2015), as well as depression (Yirmiya et al. 2015).

Many studies have shown that SD induces activated microglia, manifesting as an increased number, larger soma, fewer branches, and shorter length (Li et al. 2024; Wen et al. 2024). Numerous earlier studies indicate that the activation of microglia triggers an intense cytokine storm linked to inflammation (Ahmed et al. 2021; Li et al. 2024; Liu et al. 2024a).

Microglial activation states are categorized into two types: the M1 state, characterized by pro-inflammatory markers such as TNF, IL-1β, IL-6, and IL-18 (Tuan et al. 2023), and the M2 state, associated with anti-inflammatory factors including Arg1, BDNF, and IL-10 (Guo et al. 2022; Ji et al. 2018). It is important to note that this M1/M2 dichotomy, while useful for illustrative purposes, represents a simplification of in vivo conditions. In vivo, microglia display a vast spectrum of dynamic and context-dependent activation states that extend beyond this binary classification (Casella et al. 2016; Hirbec et al. 2019). Huan Liu et al.’s report shows that 48 h SD increased the expression of M1 microglia-associated factors while decreasing the expression of M2 microglia-­associated factors in the hippocampus, thereby activating microglia towards the M1 phenotype and inhibiting M2 activation (Liu et al. 2024a).

These proinflammatory factors can create a chronic neuroinflammatory state that is harmful to both neurogenesis and spatial memory (Valero et al. 2017). IL-1β and TNF have been reported to inhibit memory formation and impair learning and memory function (Barrientos et al. 2006; Cunningham et al. 1996). This effect may occur through their modulation of downstream MAPK and NF-κB signaling pathways, which are important for regulating learning and memory (Datusalia and Sharma 2016).

**Fig. 3:**
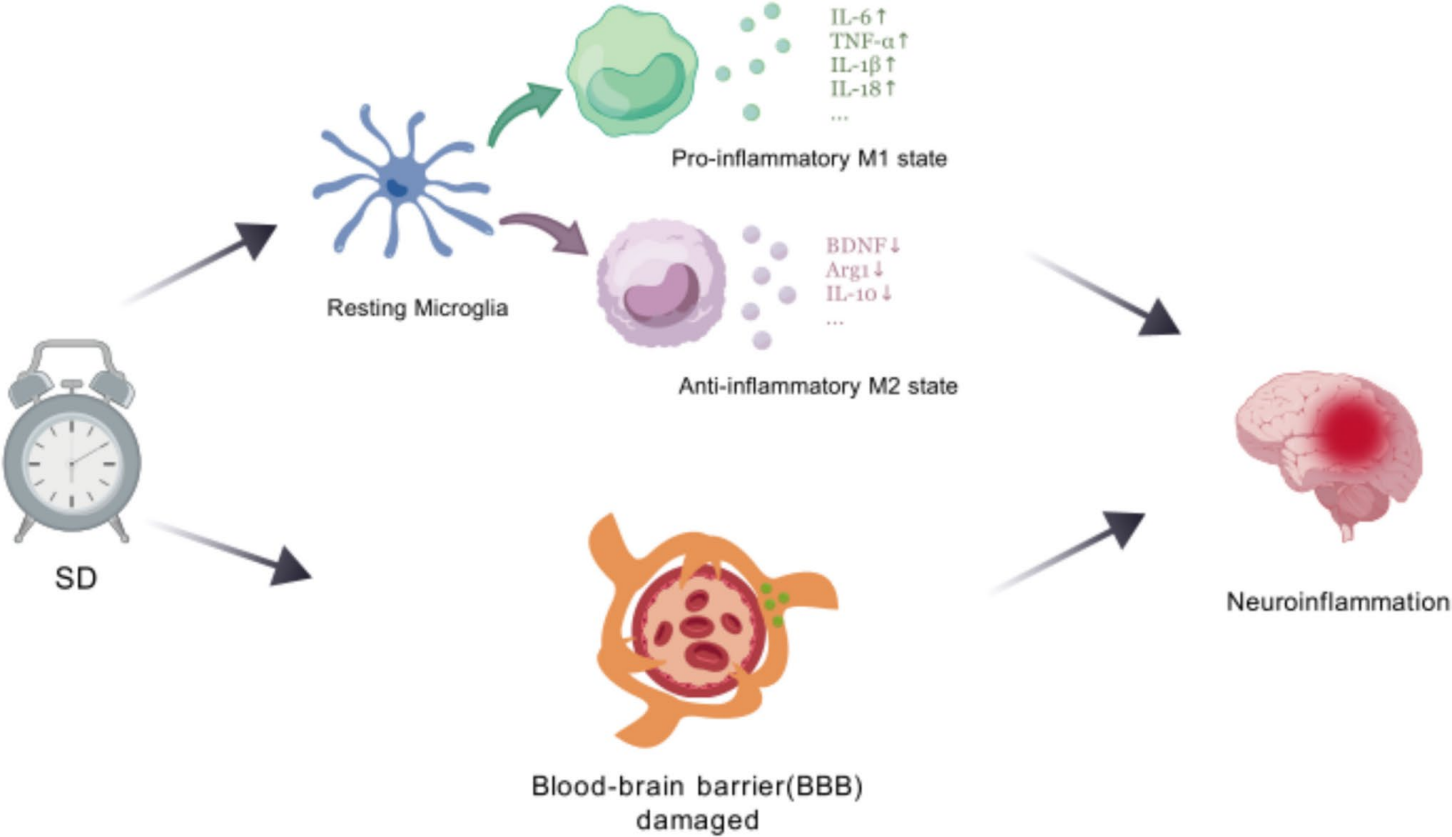
SD-induced microglial activation, BBB damage, and neuroinflammation. The M1 pro-inflammatory state releases harmful cytokines (IL-6, TNF, and IL-18), exacerbating neuroinflammation and CI. The M2 anti-inflammatory state secretes protective factors (BDNF, Arg1, IL-10), promoting neural repair. SD shifts microglia toward M1 dominance, disrupting synaptic plasticity and memory. Meanwhile, SD leads to damage of the blood–brain barrier, allowing more harmful substances to enter the cerebral circulation, which exacerbates neuroinflammation. These factors collectively result in CI

Furthermore, studies have shown that the activation of nicotinamide adenine dinucleotide phosphate (NADPH) oxidase in M1 microglia impairs P-glycoprotein (P-gp) function in brain endothelial cells. This impairment allows neurotoxic proteins to accumulate in the brain (Jiang et al. 2018) (Fig. 3).

Recent studies have consistently shown a strong link between topoisomerase 1 (TOP1) and inflammatory reactions (Ho et al. 2021; Rialdi et al. 2016; Zhu et al. 2022). A key study found that knocking down TOP1 in microglia from sleep-deprived mice raised IL-6 levels (Li et al. 2024). This indicates that TOP1 normally helps restrain IL-6 secretion from activated microglia. Losing this restraint after TOP1 knockdown contributes to neuronal dysfunction and oxidative stress, suggesting that SD-induced microglial activation and inflammation might involve increased TOP1 activity (Liu et al. 2024a, b). Separately, propofol may help switch microglia to the protective M2 phenotype, which ultimately reduced neuroinflammation, sleep architecture disturbances, neurological injury, and CI induced by acute SD. However, the long-term recovery effects of propofol on cognitive function following SD have not been assessed, and the short-term intervention outcomes cannot be directly extrapolated to CSD (Liu et al. 2024a).

Brain-derived neurotrophic factor (BDNF), produced mainly by neurons but also by microglia, is crucial for axonal growth, neuron survival, differentiation, and synaptic plasticity (Ghosh et al. 1994). Although neurons supply most BDNF, the portion released by microglia is vital in the context of memory and learning problems (Parkhurst et al. 2013).

Studies have shown that BDNF exerts multiple functions in neurobiology, such as maintaining synaptic plasticity and protecting the morphology of neuronal dendritic spines (Lu et al. 2014; von Bohlen Und Halbach and von Bohlen Und Halbach 2018), and underlies cognitive processes in the hippocampus (Liu and Nusslock 2018). It may also act directly on microglia to curb inflammation (Charlton et al. 2023). However, this anti-inflammatory effect is only seen in cell cultures and lacks validation in animal models, so its relevance in living systems is still unclear. Liu et al. demonstrated that SD decreases BDNF expression (Liu et al. 2024a); interestingly, M2-type microglia are capable of secreting BDNF (Ferrini and De Koninck 2013), which facilitates synapse formation linked to learning and modulates neuronal excitability via BDNF signaling pathways (Ferrini and De Koninck 2013; Parkhurst et al. 2013). These mechanisms are critically involved in learning and memory processes.

Extensive research indicates that suppressing excessive microglial activation, diminishing pro-inflammatory cytokines produced by microglia, and enhancing microglia-derived neurotrophic factors are pivotal for neurogenesis and cognitive performance (Prieto and Cotman 2017; Takamura et al. 2017). The drop in overall brain BDNF during SD could mean microglia lose a key protective signal, contributing to CI. Yet, it is still unclear if this BDNF decrease is directly linked to changes in microglia themselves. In the future, it will be necessary to specifically detect the BDNF content in microglia after SD to clarify this potential pathway.

SD activates microglia, triggering a cascade of inflammatory events. A hallmark of this activation is the increased release of pro-inflammatory cytokines like IL-1β and TNF(Li et al. 2024).

The blood–brain barrier (BBB), a crucial regulator of the brain’s internal environment, undergoes structural and functional deterioration from these responses (Hurtado-Alvarado et al. 2016). For example, the tight junctions between cells break down, shown by reduced and patchy expression of the key protein Claudin-5 (Voirin et al. 2020). Claudin-5 is a major tight junction protein in brain blood vessel cells that is vital for BBB integrity. When its levels fall or its organization is disrupted, the barrier becomes leakier (Chiu and Lai 2013; Greene et al. 2019).

These processes can become self-sustaining or escalate through a positive feedback loop, in which microglial activation triggers heightened inflammation and BBB impairment, further fueling the spread and intensification of microglial activity (Kang et al. 2020; Ronaldson and Davis 2020). When the BBB is impaired, it allows the infiltration of immune cells and harmful agents like toxins and pathogens into the brain. This invasion sets off worsening neuroinflammation, neuronal injury, and cell death, a process common in neurodegenerative diseases like AD and PD (Al-Bachari et al. 2020; Kerner and Roose 2016; Lee and Pienaar 2014; Lee and Funk 2023; Sweeney et al. 2018; Zolotoff et al. 2020).

Increased levels of Iba-1, a marker of microglial activation (Ito et al. 1998; Minett et al. 2016) that is also involved in cognition (Lituma et al. 2021), are seen in SF and may contribute to both BBB damage and cognitive problems (Puech et al. 2023). These alterations in BBB permeability are concomitant with a significant impairment in explicit memory function, reflecting the impact of microglia on CI from the perspective of altering blood–brain barrier permeability during SD.

### Induction of Autophagy and Cell Death of Microglia

Autophagy is an essential intracellular clearance mechanism that preserves cellular homeostasis by breaking down and reusing proteins and organelles within lysosomes (Piletic et al. 2023; Yao et al. 2021). At normal levels, it protects and repairs neurons, but excessive autophagy causes cellular stress, oxidative damage, and can lead to cell death (Li et al. 2021; Liu et al. 2024b). The protein S100A8 is a key driver of SD-related damage to hippocampal neurons in mice. After CSD, S100A8 is found mainly in microglia, not in astrocytes or neurons in the hippocampus (Xiong et al. 2024). The elevated S100A8 levels likely contribute to CSD-triggered CI through regulation of microglial activation pathways. Hippocampal tissues of CSD-exposed mice exhibited significant augmentation of double-membrane autophagosome aggregation, correlating with observed CI. Notably, genetic suppression of S100A8 substantially diminished autophagosome formation, effectively rescued hippocampal neuropathology, and consequently alleviated CSD-induced cognitive dysfunction, which suggests microglia impact cognitive function after SD by modulating autophagy mediated by the level of S100A8. However, the benefits of deleting S100A8 may come from its effect on several combined pathways, not just one, which needs more study (Xiong et al. 2024).

Furthermore, in the study by Xiong et al., TUNEL staining revealed that CSD significantly increased cell death in the hippocampal region, while S100A8 knockdown effectively suppressed this cell death process. This protective effect was accompanied by in vitro findings that S100A8 knockdown reduced apoptosis in rapamycin-stimulated BV2 microglial cells (a cell line derived from mouse microglia), specifically manifested as significantly suppressed expression of caspase-3 and Bax, along with upregulated expression of Bcl-2. As previously described, S100A8 is primarily localized in microglia. These findings collectively indicate that CSD induces CI by promoting microglial cell death, a process potentially associated with ROS production and the MAPK signaling pathway (Xiong et al. 2024). The S100A8/A9 complex binds to the RAGE receptor, which then triggers the phosphorylation of p38 and p44/42 MAPK and activates NF-κB. S100A8 also boosts the production of reactive oxygen species, which damages mitochondria. Cytochrome C, Smac, and HtrA2 are concomitantly released from mitochondria into the cytoplasm (Wang et al. 2018). Cytochrome C combines with Apaf-1 and procaspase-9 to form an “apoptosome,” which activates caspase-9. This activates the final “executioner” enzymes, caspase-3 and caspase-7. Meanwhile, MAPK phosphorylation makes cells more prone to die by increasing pro-death proteins (like Bax) and blocking survival signals (like Bcl-2 and Akt) (Ganguly et al. 2023).

### Disruption of Synaptic Plasticity

Insufficient sleep has been shown to heighten neural hyperactivity through oxidative damage, potentially resulting in neurodegeneration, impaired neuronal physiology, compromised synaptic flexibility, and induced cellular cell death, ultimately undermining synaptic efficacy (Kincheski et al. 2017). Microglia interact with neurons to maintain brain balance and work together to support the growth of new neurons and memory formation (Cornell et al. 2022).

#### Synaptic Pruning

SD disrupts cognitive function by impairing memory consolidation, which is tightly linked to synaptic plasticity deficits in synaptic circuits, in which microglia play a very important role (Tuan and Lee 2019; Zhan et al. 2014). One key job of microglia is “synaptic pruning”, that is, removing unnecessary neural connections, which is essential for properly shaping brain circuits and their plasticity (Schafer et al. 2012). Microglia are the resident phagocytes of the central nervous system (CNS). During wakefulness, microglia usually display an elongated morphology characterized by thin, ramified, and lengthy projections (Wang et al. 2023b). They periodically reach out with their extensions to momentarily contact and monitor the functional state of synapses in an activity-dependent manner (Tremblay et al. 2010; Wake et al. 2009), sense neuronal activity, and clear neuronal debris after injury and cell death (Tay et al. 2017; Tremblay et al. 2010; Wake et al. 2009), and contribute to developmental synaptic pruning in the healthy brain (Paolicelli et al. 2011; Schafer et al. 2012; Sipe et al. 2016). Just 3 h of SD can change their shape dramatically. They shift to a rounder, “amoeboid” form with a larger cell body and shorter, fewer branches, ready for phagocytosis (Wang et al. 2023b). This change boosts their ability to engulf material. Microglia-mediated synaptic pruning mainly depends on surface receptors and membrane molecules, such as CX3CR1, TREM2, and C1q, which are essential for recognizing, engulfing, and targeting synapses (Lan et al. 2022; Lee and Chung 2019; Miyamoto et al. 2013).

In the CNS, the fractalkine receptor (CX3CR1), a key protein involved in synaptic pruning, is predominantly found on microglia and functions as an “eat-me” signal molecule (Lee and Chung 2019). It controls the microglial skeleton and movement to enable pruning (Ball et al. 2022; Kozareva et al. 2019; Lee and Chung 2019; Paolicelli et al. 2011). Lower CX3CR1 levels disrupt normal pruning and lead to a buildup of dendritic spines in certain brain areas (Fernández de Cossío et al. 2017). Microglial CX3CR1 expression appears to be downregulated in sleep-deprived mice, resulting in suppressed synaptic pruning via this pathway and ultimately contributing to CI (Wang et al. 2023b).

#### Synapse Engulfment

Memory formation relies on changes in synaptic connections. A healthy balance between creating new dendritic spines and pruning old ones is vital for cognition (Choi et al. 2018). Increasing evidence indicates that various forms of SD result in the loss of dendritic spines in the hippocampus (Bolsius et al. 2022; Havekes et al. 2016). Normally, microglia help wire the brain correctly by phagocytosing extra synapses (Paolicelli et al. 2011; Schafer et al. 2012; Tremblay et al. 2010), clearing surplus myelin sheaths, and stopping abnormal myelination of cell bodies (Hughes and Appel 2020). Na Li et al. indicates that CSD triggers microglial activation and enhances their ability to engulf synaptic components (Li et al. 2023). A hallmark of neuronal injury, synaptic impairment is frequently characterized by reduced dendritic spine density and widened synaptic clefts. Pretreating mice with short-chain fatty acids reduced both CI and microglial activation and pruning caused by SD (Li et al. 2023). This indicates that excessive synaptic phagocytosis by microglia during SD could lead to CI. However, it is not yet proven that SCFAs work specifically by controlling microglial synapse eating, and direct causal evidence for this mechanism is missing.

The triggering receptor expressed on myeloid cells 2 (TREM2), an immunoglobulin superfamily component, is an innate immune receptor predominantly found in microglia (Karch and Goate 2015). It plays a vital role in neurodevelopmental processes such as synaptic pruning and the establishment of proper brain connectivity, both of which are crucial for normal behavioral functioning. Loss of TREM2 has been shown to disrupt neuronal circuitry and impair social interaction abilities (Filipello et al. 2018).

CD33, alternatively referred to as Siglec-3, is an immune-modulating receptor on microglia linked to AD risk. In the brain, it’s found mostly on microglia, and its levels rise significantly in these cells during diseases like AD (Jiang et al. 2014). In AD’s pathology, CD33 is known to block the phagocytic activity that TREM2 triggers in microglia (Griciuc et al. 2019). In the CSR mouse model, hippocampal CD33 protein expression was markedly reduced, whereas TREM2 levels showed a significant upregulation. Microglial phagocytosis also increased during this sleep restriction. These findings suggest that lower CD33 levels may remove a brake on TREM2, leading to excessive synapse clearance by microglia and, ultimately, CI (Tan et al. 2023b). However, the study did not employ electron microscopy to observe synaptic fragments within microglia, resulting in a lack of direct morphological evidence for synaptic phagocytosis.

On the other hand, mice lacking the CX3CR1 receptor are protected from the excessive microglial activation caused by SD. After SD, these mice had lower levels of phagocytosis signals and IL-1β, and higher levels of anti-inflammatory markers including Arginase-1 (Arg1) and interleukin-4 (IL-4). These findings demonstrate that CX3CR1 regulates microglial hyperactivation and excessive synaptic pruning triggered by SD, with CX3CR1 deficiency mitigating these effects and ultimately enhancing cognitive performance (Xin et al. 2021).

**Fig. 4:**
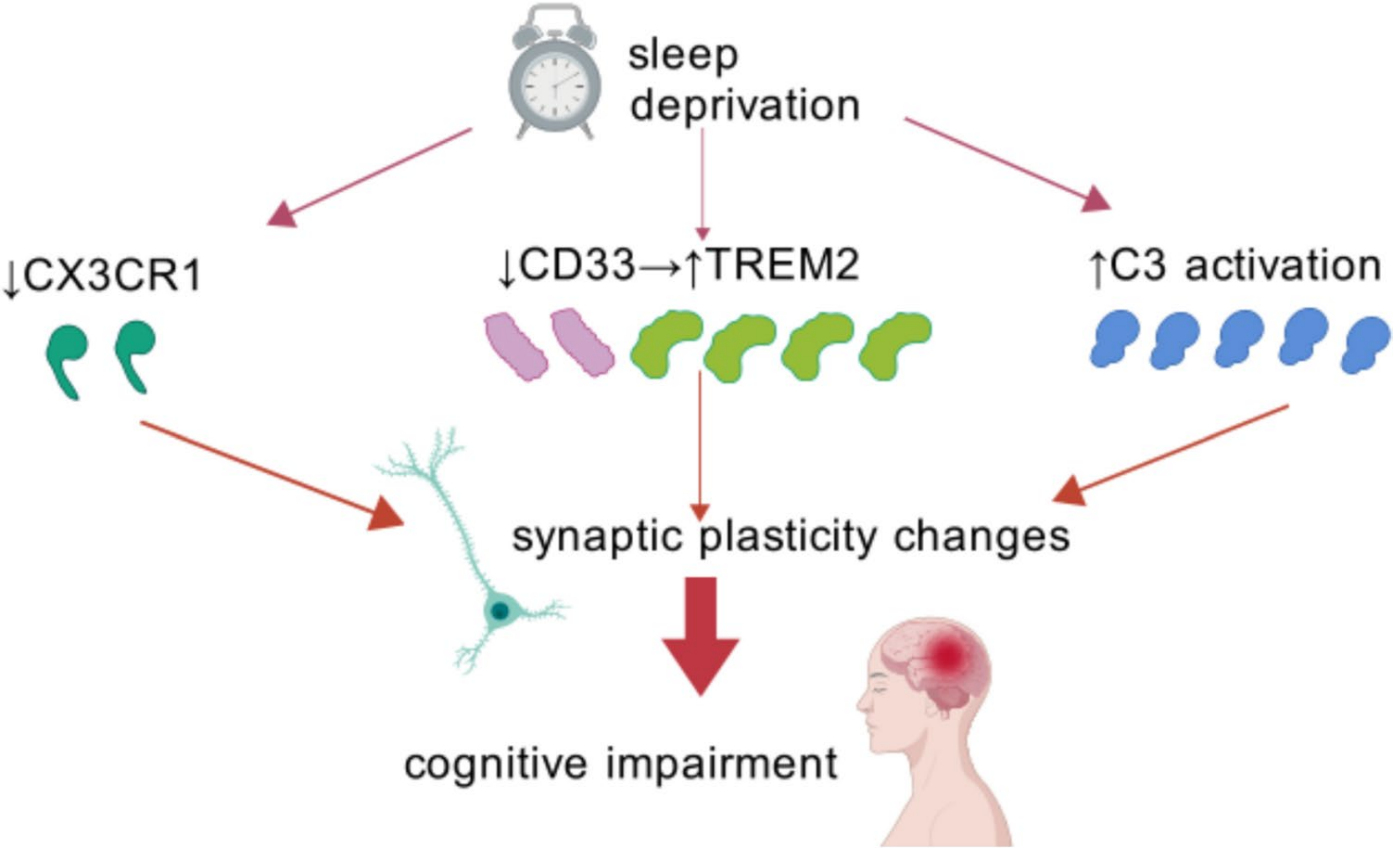
Synaptic pruning mechanisms in SD-induced CI. This figure summarizes three ways SD disrupts synaptic plasticity via microglia: CX3CR1 downregulation impairs physiological synaptic pruning, leading to dendritic spine accumulation. CD33 suppression may enhance TREM2-mediated excessive phagocytosis of synapses. Complement C3 activation promotes aberrant microglial engulfment of synapses. Each pathway converges on synaptic loss and cognitive deficits. Arrows indicate activation/inhibition relationships, with color-coding for microglial states (M1/M2). Key molecules (CX3CR1, CD33, TREM2, C3) are highlighted

As a key mediator, complement C3 induces synaptic pruning by microglia. Microglial phagocytosis is facilitated by complement proteins C1q and C3 that mark superfluous synapses, as well as by microglia-expressed complement receptors involved in phagocytic clearance (Stevens et al. 2007). As a key element of the complement system, C3 binds to cellular debris and can directly activate microglial C3 receptors, inducing elimination by phagocytosis. SD can trigger C3 activation (Bellesi et al. 2017). CSR, which activates microglia, also increased C3 levels and microglial phagocytosis. This fits with the idea that activated microglia use the complement pathway to over-engulf synapses during sleep loss (Liu et al. 2023) (Fig. 4).

## The Protective Role of Microglia in SD-Induced CI

Genetic and functional studies show microglia are involved in neurodegenerative diseases, but how much they protect versus harm is still discussed (Colonna and Butovsky 2017; Li and Barres 2018). However, the dual role of microglia in the CNS is well-recognized (Guo et al. 2022; Konishi and Kiyama 2018; Peng et al. 2022). For instance, in models of neurodegeneration, microglia secrete not only neurotoxic substances but also factors that protect neurons (Wes et al. 2016).

The protein STING, found in cells including microglia, is key for defending against pathogens. In innate immunity, the STING-TBK1-IRF3 pathway is a major signaling route and is involved in many diseases like autoimmunity and cancer (Corrales et al. 2016; Li et al. 2019; Woo et al. 2014). Triggering receptor expressed on myeloid cells 2 (TREM2), a key innate immune receptor involved in microglial regulation, is predominantly found in microglia (Poliani et al. 2015). Activation of TREM2 has been shown to alleviate neuroinflammation and enhance cognitive function in mice following surgery (Han et al. 2022). Earlier work found TREM2 levels rise after CSR (Tan et al. 2023a; Wang et al. 2024). Yue Wang et al. found that administering a STING agonist into the hippocampus alleviates CSR-triggered neuroinflammation, CI, and neuronal damage through the STING-TBK1-IRF3 pathway by increasing TREM2 expression and shifting microglia from a pro-inflammatory to an anti-inflammatory phenotype (Wang et al. 2024).

The α7 nicotinic acetylcholine receptor (α7-nAChR) helps control immune responses and oxidative stress in the brain and body (Ren et al. 2017). Strong evidence shows this receptor is important for managing excitatory signals, enhancing learning and memory, and supporting overall cognition (Shen and Wu 2015). Activation of PI3K/AKT downstream of α7-nAChR suppresses glycogen synthase kinase-3β (GSK-3β) (Borovikova et al. 2000; Parrish et al. 2008), leading to upregulation of the antioxidant enzyme HO-1 and reduction of pro-inflammatory cytokine release (Brugge et al. 2007; Charpantier et al. 2005; McCubrey et al. 2014). Multiple central nervous system diseases, from AD and PD to depression and ADHD, are linked to problems in α7-nAChR signaling (Han et al. 2014; Taly et al. 2009). More and more studies have found the expression of α7-nAChR on the surfaces of microglia (De Simone et al. 2005; Shytle et al. 2004). Following CSD, α7-nAChR levels in microglia decreased, accompanied by elevated pro-inflammatory cytokines, diminished anti-inflammatory mediators and antioxidative enzymes, as well as suppressed PI3K/AKT/GSK-3β signaling. A drug that activates α7-nAChR (PHA-543613) reversed this weakened signaling (Xue et al. 2019). Therefore, microglia may inhibit the inflammatory response in SD through the regulation of α7-nAChR in the PI3K/AKT/GSK-3β pathway, which plays an important role in protecting cognitive function. Nevertheless, the alterations and functional impact of α7nAChR in the CNS following SD have yet to be elucidated.

## Discussion

This review has combined evidence from the last 20 years showing the many ways microglia are involved in SD-related CI. These include problems with neurotransmitters, amyloid-beta buildup, brain inflammation, faulty autophagy, cell death, and disrupted synaptic plasticity. Although progress is significant, turning these discoveries into treatments requires a honest look at the current evidence’s limits and a clear plan for future work.

### Integrated Pathogenic Network in SD-Induced CI

The pathways we discussed do not work alone. They form a connected network, with microglial activation, triggered by sleep loss, at the center. We can think about their importance and how they interact like this:

Neuroinflammation seems to be a central and major driver, made worse by nearly every other pathway. SD-induced microglial activation promotes a pro-inflammatory state, releasing cytokines (e.g., IL-1β, TNF, IL-6) that directly impair synaptic function and neuronal health. Faulty synaptic plasticity is a key mechanism that directly causes CI. It is strongly affected by neuroinflammation (via cytokine release), aberrant synaptic pruning (via CX3CR1, TREM2, and C1q/C3), and excitotoxicity. The loss of protective factors like BDNF further contributes. Excitotoxicity and neurotransmitter problems act as both cause and effect. Activated microglia can start it by releasing too much glutamate, which harms neurons, hurts synapses, and feeds inflammation. Aβ deposition and tau pathology (implied) represent a significant amplifying loop. SD disrupts clearance, leading to Aβ accumulation. Aβ can then further activate microglia, perpetuating neuroinflammation and potentially influencing synaptic pruning. The APOE4 genotype synergizes strongly with SD here. Autophagy and cell death (e.g., via S100A8) represent downstream executioner pathways triggered by intense inflammatory and stress signals, leading to overt cellular damage and loss. BBB disruption is a result, often of bad inflammation. It lets inflammatory signals from the body into the brain, which then worsens microglial activation and harms neurons more.

### Methodological Considerations and Future Directions

The compelling narrative of microglial involvement in SD-induced CI, which we and others present, needs to be balanced with a critical look at how the supporting evidence was gathered.

#### Field-Wide Limitations

Although the role of microglia in SD-induced CI has gained increasing attention, current research still faces several important limitations that need to be addressed in future work. Most animal studies use young male mice or rats. They ignore how age and sex, which affect AD and sleep disorder risk, change inflammatory responses and disease progression. At the same time, human studies are not only scarce but also typically involve small sample sizes (often n = 6–20), predominantly focusing on healthy young males, which limits their representation of clinical populations such as individuals with CSD or those at high risk for AD.

For animal models, SD is usually short-term (24–72 h), not matching long-term human sleep problems like insomnia. Common AD mouse models quickly develop amyloid plaques but often lack the tau tangles seen in most human AD cases. Moreover, in vitro studies widely employ immortalized cell lines (e.g., BV2 microglia), which may not fully recapitulate the biological characteristics of primary or human microglia.

For mechanisms, most studies only describe linked events (e.g., “sleep loss leads to more TREM2, which causes inflammation”) without finding the key middle-step molecules that connect them. This leaves multiple “black boxes” in the mechanistic cascade, and there is often insufficient distinction between pathogenic species (e.g., phosphorylated/oligomeric tau, soluble/insoluble Aβ).

Conclusions rely heavily on animals but not on checking human brain tissue, spinal fluid, or stem-cell models. We especially lack long-term human studies connecting chronic sleep problems to AD development, making cause-and-effect hard to prove. In terms of therapeutic strategies (such as targeting DRD2 or α7-nAChR), there is an absence of systematic assessment of long-term safety and efficacy in humans.

Some study designs have flaws, like not “blinding” the researchers during analysis, which can add bias. Assessment metrics are often narrow (e.g., focusing solely on synaptic structure without functional electrophysiological validation such as LTP), making it difficult to comprehensively establish structure–function relationships.

Therefore, future work needs better, more human-relevant models, stronger proof of cause-and-effect mechanisms, steps toward human testing, and improved study designs to make findings more reliable and widely applicable.

#### Future Research Directions

To further investigate the role of microglia in SD-induced CI, future studies should focus on developing more clinically relevant experimental models that incorporate both female and aged animals to explore age- and sex-specific effects. This includes establishing models such as chronic sleep restriction combined with AD risk genotypes or sporadic AD backgrounds. In vitro studies should prioritize the use of human primary cells or iPSC-derived microglia. Mechanistically, it is essential to integrate techniques such as co-immunoprecipitation (Co-IP), CRISPR-based screens, and multi-omics approaches (e.g., transcriptomics and proteomics) to identify critical missing links in signaling pathways, such as adaptor proteins between CD33 and TREM2 or downstream targets of BDNF, and to precisely define key pathogenic entities, including specific Aβ oligomers and phosphorylated tau isoforms.

Furthermore, human research should be strengthened through large-scale longitudinal cohort studies that examine the relationships between chronic sleep patterns, cerebrospinal fluid/PET biomarkers (Aβ, tau, neuroinflammation), and CI/AD incidence. Findings from animal models require confirmation in human brain tissue after death. Targeted pilot clinical trials should also be initiated. For example, we could test existing drugs that activate DRD2 or α7-nAChR receptors in people with poor sleep and early memory concerns.

Experimental rigor must be enhanced through stringent implementation and reporting of blinding and allocation concealment, alongside integrated multi-level assessments combining molecular, cellular (including structural and functional electrophysiology), and behavioral (cognitive and affective) readouts. To move toward treatments, work should focus on: creating better ways to deliver drugs to the brain (e.g., nanoparticles for TREM2 drugs), testing combined approaches like better sleep habits plus anti-inflammatory medicine, and creating tailored strategies for different disease stages, e.g., preclinical AD. A key unanswered question is exactly how S100A8 drives too much microglial autophagy after sleep loss. Understanding this process is an important goal for future work.

### Conclusion

In conclusion, microglia emerge as pivotal mediators in the detrimental cognitive consequences of SD, orchestrating a complex cascade of events spanning neuroinflammation, excitotoxicity, impaired clearance, and synaptic dysfunction. While the outlined pathways provide a framework for understanding this process, critical knowledge gaps remain.

We do not know the order or relative importance of these mechanisms during different types and lengths of sleep loss. We also lack a clear mechanistic picture of how SD shifts microglia from their normal state to either a protective or a harmful one. Crucially, the vast majority of mechanistic evidence is derived from rodent models, whose translatability to human sleep disorders and neurodegenerative diseases requires rigorous validation, as detailed above. Furthermore, whether strategies targeting microglia actually work in people, when to use them, and if they are safe long-term, are questions hardly studied in humans. Finally, microglial activation might be protective early on (e.g., clearing Aβ) but turns harmful in the later stage (excessive synaptic pruning). A critical unknown is what triggers this switch from helpful to harmful, and how we might control it. In the future, our research should focus more on this issue and find the balance within it to better guide the clinical translation of drugs.

Closing these gaps will require a multidisciplinary approach: better animal models that reflect human disease diversity, advanced brain imaging and biomarkers in people, detailed studies of microglial signaling, and finally, well-designed clinical trials. Bridging the divide between the compelling biology observed in the laboratory and the urgent need for effective interventions in sleep-disturbed populations represents the central challenge and opportunity for this field.

## Data Availability

No datasets were generated or analysed during the current study.
